# Micronutrients and the Periodontium: A Narrative Review

**DOI:** 10.7759/cureus.81694

**Published:** 2025-04-04

**Authors:** Dhruvi Doshi, Santosh Kumar, Bhavin Patel, Dipak Chaudhari, Shirishkumar Patel, Tanvi Hirani, Hiren H Patadiya, Isha R Bhingradia, Mainul Haque

**Affiliations:** 1 Periodontology and Implantology, Karnavati School of Dentistry, Karnavati University, Gandhinagar, IND; 2 General Dentistry, My Dental Southbridge PLLC, Southbridge, USA; 3 Pharmacology and Therapeutics, National Defence University of Malaysia, Kuala Lumpur, MYS; 4 Research, Karnavati School of Dentistry, Karnavati University, Gandhinagar, IND

**Keywords:** alpha tocopherol, ascorbic acid, calciferols, cobalamin (b12), macronutrients, micronutrients, minerals, periodontal disease, phylloquinone, retinol

## Abstract

This is a comprehensive narrative assessment of micronutrient relevance for periodontal health. The periodontium is a crucial and intricate structure that supports the tooth within the alveolar bone. Composed of four tissues with distinct embryological origins- the alveolar bone, cementum, gingiva, and periodontal ligament - the periodontium plays a fundamental role in maintaining dento-alveolar function and homeostasis. Periodontitis is gaining attention due to its widespread occurrence worldwide. This inflammatory condition disrupts the balance between the host immune response and microbial virulence factors. Connective tissue production and host defenses require proteins for periodontal health. Vegetable proteins support periodontal health by promoting tissue repair and immune function, while excessive dietary fats can exacerbate inflammation and increase the risk of periodontal disease (PD). Inflammation is further compounded due to dietary animal fats, which contribute to the risk of PD. Omega-3 fatty acids are well-known for their anti-inflammatory effects, which help reduce periodontal inflammation. Adequate intake of essential minerals and vitamins is required for maintaining periodontal health. Vitamins K, D, and A are crucial in maintaining oral epithelial integrity, facilitating bone development, and supporting overall tissue maintenance. Inadequate calcium (Ca²⁺) and magnesium (Mg²⁺) levels have been associated with severe PD. Antioxidants such as vitamin C help alleviate inflammation in periodontal tissues. As recognized by dental professionals, nutritional status plays a considerable role in an individual's risk of developing PD.

## Introduction and background

Periodontitis, a chronic inflammatory condition, harms the supporting tissues [1]. If left untreated, periodontitis can cause tooth loss, leading to difficulties in chewing, changes in dietary habits, and potential nutritional imbalances [1,2]. This chronic inflammatory disorder is primarily triggered by specific periodontal pathogens, such as Aggregatibacter actinomycetemcomitans and Porphyromonas gingivalis [3,4]. The main factor contributing to tooth loss is the deterioration of the periodontal ligament and alveolar bone, which characterizes the condition's progression [3]. Extensive research has established connections between periodontitis and several systemic health issues, including complications during pregnancy, cardiovascular diseases, diabetes mellitus, gestational diabetes, atherosclerosis, and rheumatoid arthritis [5]. Nutritional factors have been connected to several chronic inflammatory periodontitis-related diseases [6].

A balanced diet and good nutritional status are essential for fostering growth and tissue repair and are crucial in reducing the risk of many diseases [7]. The nutritional state considerably affects how periodontal disease (PD) advances and how well the periodontal tissues recover [3,8]. Low serum or plasma micronutrient levels, which are connected to periodontitis, can be influenced by lifestyle choices, diet, and hereditary factors related to nutrition [6,9]. Vitamins serve as catalysts in metabolism, aiding the body in converting proteins, fats, and carbohydrates into energy, supporting growth, and maintaining cells [10].

Vitamins may be obtained through diet and supplements or produced by our body or gut microbiota [11]. Vitamins are crucial in oral and overall health, and an imbalance can result in malnutrition [10]. Scurvy, rickets, pellagra, and beriberi are among the systemic problems that can result from severe vitamin deficits [11]. Nutrition is the biological process by which living organisms break down dietary components to produce vital substances necessary for growth, development, survival, and reproduction [12]. Nutrients are classified into two groups: micronutrients and macronutrients [12,13]. Macronutrients, including proteins, lipids, and carbohydrates, supply the energy required for cellular functions essential to daily activities [12,14]. Micronutrients, such as minerals and vitamins, are critical for metabolism, development, growth, and general physiological health, even though they are required in lesser amounts [12,14].

Micronutrients refer to vitamins, minerals, and trace elements [15]. The body needs them in daily amounts of less than 100 mg, and they are thought to play a role in PD [15]. Inadequate mineral and vitamin levels have been linked to triggering chronic and degenerative processes and cellular aging, as their deficiency continuously disrupts metabolic processes [14]. Vitamin C and B-complex vitamins are water-soluble, while vitamins A, D, E, and K are fat-soluble, meaning they dissolve in fats and oils rather than water [14,15].

Objectives of the study

This study investigates the role of essential micronutrients - vitamins D, A, E, and C, along with the minerals magnesium (Mg²⁺), zinc (Zn²⁺), and calcium (Ca²⁺) - in periodontal health. The review also evaluated deficiencies, optimal levels, and supplementation benefits to improve PD prevention, management, and therapeutic strategies for better oral health. Furthermore, it examines their impact on inflammation control, tissue regeneration, and bone density.

## Review

Materials and methods

**Figure 1:**
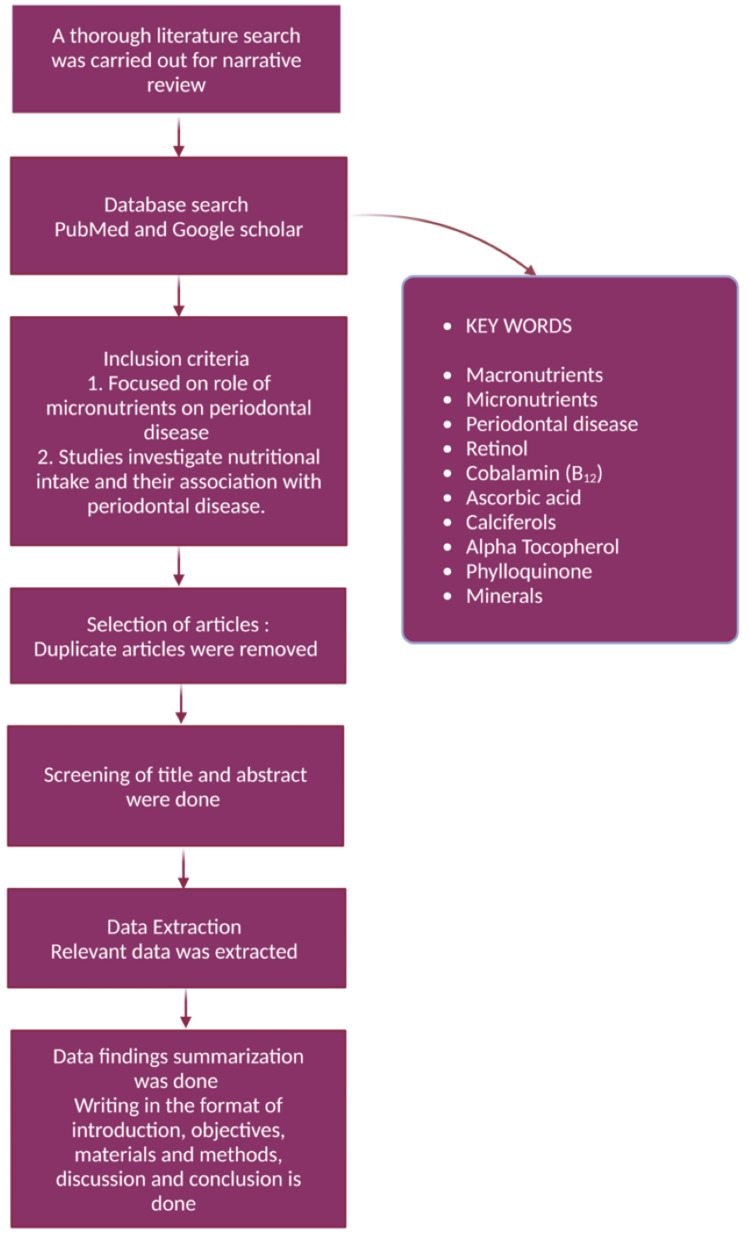
Methodology of this narrative review. Notes: This image was created with BioRender premium version [16], with an agreement license number GQ2825QVWP. Illustration Credit: Dhruvi Doshi

This study was a comprehensive narrative review that was carried out using PubMed and Google Scholar databases to examine the impact of micronutrients on PD. The inclusion criteria were (1) studies focusing on micronutrients’ impact on PD and (2) research investigating nutritional intake and its association with periodontal health. Duplicate articles were removed, and titles and abstracts were screened for relevance. Only studies meeting the criteria were selected for further analysis. Data extraction was carried out, focusing on the effects of vitamins C, B₁₂, A, D, E, and K and essential minerals to maintain periodontal health. Figure 1 outlines the structured approach used in this study. The structured methodology of this review paper offers a clear and comprehensive understanding of the influence of micronutrients on PD based on existing scientific evidence.

Review of literature

Vitamins

Vitamin A: Retinol is a type of fat-soluble vitamin belonging to a class of substances that includes beta-carotene and other carotenoids [5,17,18]. Alpha-carotene, lutein, beta-carotene, cryptoxanthin, lycopene, and zeaxanthin are among the pigments found naturally in the environment known as carotenoids that give plants and other animals their color [19]. Foods rich in vitamin A include cod liver oil, broccoli, eggs, sweet potatoes, carrots, leafy greens, and bell peppers [13]. According to the UK National Health Service, men between the ages of 19 and 64 need 0.7 mg of vitamin A daily, while women in the same age range require 0.6 mg per day [20,21].

Vitamin A can enhance the osteogenic differentiation, proliferation, and migration of human periodontal ligament cells, indicating its potential to support periodontal tissue regeneration [22]. Vitamin A maintains mucosal cells' structural and functional health. Additionally, it is indispensable for the optimal activity of B and T lymphocytes, which play a vital role in strengthening the body's immune defense [23,24]. Inflammatory cytokines are key regulators in developing, healing, and regenerating PD. Meanwhile, vitamin A and its metabolites act as biomolecules that modulate inflammation and influence cellular pluripotency and inflammatory responses [25]. A nutritional survey conducted across populations in Ethiopia, Vietnam, Lebanon, Alaska, Ecuador, Chile, Colombia, and Thailand found that vitamin A deficiency was commonly associated with a higher incidence of PD [15]. Consuming at least 7.07 mg of beta-carotene per day has been linked to decreased sites with a probing depth greater than 3 mm after scaling and root planing [20].

Vitamin B: The vitamin B complex family is composed of thiamine (B_1_), riboflavin (B_2_), niacin (B_3_), pantothenic acid (B_5_), pyridoxine, pyridoxal, and pyridoxamine (grouped as B_6_), biotin (B_7_), folate (B_9_), and cobalamin (B_12_) [20]. The UK National Health Service advises adults to consume 200 µg of vitamin B_9 _daily, sourced from leafy greens and fortified cereals, along with 1.5 µg of vitamin B_12_, which can be obtained from fortified cereals, meat, and fish [20,21].

Thiamine supports healthy muscle and nerve function, especially converting glucose into energy [5,26]. Insufficient thiamine intake significantly impairs cellular energy production, leading to early signs such as fatigue, peripheral neuropathy, nausea, constipation, and depression [27]. A persistent lack of thiamine may lead to severe health complications over time, which affect the cardiovascular, nervous, and muscular systems. Conditions such as beriberi, often linked to high-carbohydrate diets low in thiamine, and Wernicke-Korsakoff syndrome, frequently observed in chronic alcoholics due to inadequate nutrition, are associated with this deficiency. Thiamine deficiency commonly occurs alongside riboflavin deficiency, leading to oral health issues, such as angular cheilosis, glossitis, stomatitis, taste impairment, burning tongue sensation, and heightened sensitivity of the oral mucosa [12]. Because it is present in sufficient levels in grains, eggs, meat, and milk, riboflavin is necessary for growth and development, particularly for muscles and hair, and deficiencies are rare [5].

Regular enzymatic activity and the formation of collagen structures during wound healing depend heavily on niacin [5,28]. Pellagra is marked by the severe triad of diarrhea, dermatitis, and depression, while a niacin deficiency can lead to dementia, dermatitis, diarrhea, weight loss, depression, and glossitis [29]. This deficiency may arise from physiological conditions such as Hartnup disease (which is a sporadic, heritable metabolic ailment categorized by compromised absorption of specific amino acids, principally tryptophan, causing various issues, e.g., neurological, including psychiatric difficulties, photosensitive rash) [30], malignant carcinoid syndrome, or drug therapies, such as isoniazid [27]. High cholesterol levels can be treated using niacin [12]. A separate study found that consistently consuming more than 2 g of vitamin B_^3^_ (niacin) daily may lead to liver toxicity and even liver failure [12,29]. Pantothenic acid is essential for energy metabolism, facilitating the breakdown of fats, carbohydrates, and proteins. Additionally, it aids in converting hydroxyproline to proline, a crucial amino acid for collagen synthesis, and stimulates cellular growth and repair [5]. 

Pyridoxine, or vitamin B_6,_ is essential for metabolizing amino acids [5,31,32]. Pyridoxal, pyridoxine, and pyridoxamine are the three primary compounds from vitamin B_6_ [12]. Their key function is to aid in heme synthesis. Carpal tunnel syndrome has also been treated with vitamin B6, albeit its long-term efficacy varies [12]. Protein intake and the quantity of vitamin B6 required in the diet are correlated; for healthy adults, this range usually falls between 1.4 and 2.0 mg per day [27]. Numerous foods contain biotin, which the gut microbiota produces [5]. It is essential for metabolizing carbohydrates, fats, and proteins; aids in hormonal synthesis and function; and plays a key role in erythropoiesis [27].

Vitamin B_12 _and folate, water-soluble members of the B-complex vitamin group, remain essential for forming blood cells and numerous physiological functions, including collagen production [33]. Vitamin B_12_, also called cyanocobalamin, and folic acid (FA) are interconnected B-complex vitamins crucial in supporting red blood cell production in the bone marrow. A lack of either vitamin can lead to profound anemia [34]. FA is a crucial micronutrient involved in the conversion of homocysteine to methionine, methylation processes, and purine and pyrimidine synthesis, all of which are essential for preventing and repairing DNA damage; additionally, it demonstrates anti-mutagenic properties by inducing apoptosis in dysplastic cells with damaged DNA via p53 (a critical tumor suppressor protein, is stimulated by different cellular stresses such as DNA damage, hypoxia, and oncogene stimulation activation and Bcl-2 gene suppression [35,36] (possesses captious anti-apoptotic mode of action, predominantly encompasses Bcl-2 protein impeding the liberation of pro-apoptotic elements from mitochondria, consequently averting apoptosis) [5]. Pernicious anemia is the primary condition linked to vitamin B_12 _deficiency [12]. Pernicious anemia is characterized by weakness, paleness, changes in taste and eyesight, tingling or numbness, increased frequency of urination, sadness, mood swings, and, in severe cases, psychotic episodes [37]. According to a study by Zong et al., low serum vitamin B_12_ levels are associated with a higher likelihood of developing periodontal lesions [38]. Patients receiving B-complex vitamin supplementation showed more remarkable improvement in clinical attachment levels than those given a placebo [15,34]. In individuals with chronic periodontitis, a recent study found a correlation between decreased serum vitamin B_12 _levels and elevated clinical attachment loss (CAL) [17].

Vitamin C: During the 18th century, scurvy, a condition resulting from vitamin C deficiency, was common among sailors and often manifested as bleeding gums and loosened teeth [6]. Vitamin C's capacity to cure scurvy led to its designation as ascorbic acid (AA) [12]. Approximately 10% of the general population has been found to have mild vitamin C deficiency, defined as serum levels below 24.8 μM/L. However, this condition is relatively rare in wealthier nations. Blood AA levels should be between 23 and 50 μM, but scurvy results when they fall below 11 μM [17,39]. Collagen formation, immunological response, and antioxidant defense against free radicals all depend on vitamin C [40]. In animals, it functions as a reducing agent, shielding cells from oxidative harm, and as a cofactor for enzymes involved in tyrosine metabolism, collagen synthesis, and norepinephrine production [17,40]. Two clinical studies suggest that consuming more vitamin C-rich fruits, including kiwis, peppers, and grapefruits, may help alleviate inflammation in the gums and periodontal tissues [15,41]. Those with adequate serum levels of vitamin C exhibited less attachment loss than those with a vitamin C deficiency [15,42].

**Figure 2:**
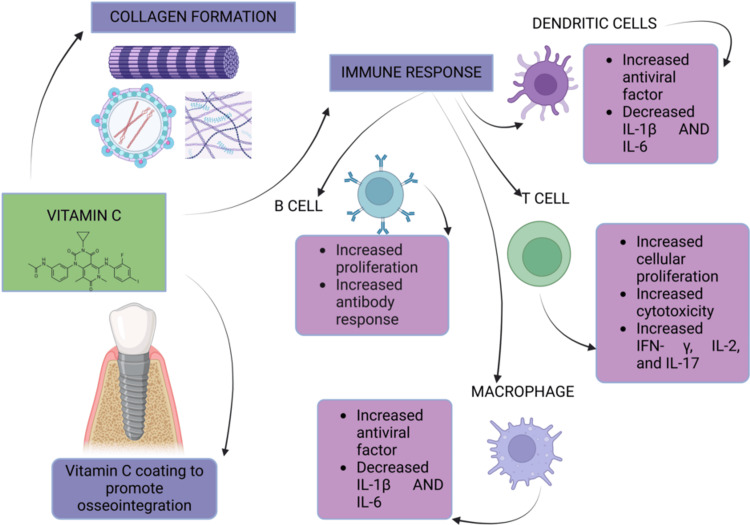
Importance of vitamin C. Notes: This image was created with BioRender [16] premium version, with an agreement license number RB28297DAI. Illustration Credit: Dhruvi Doshi

Because of its beneficial effects on periodontal well-being, vitamin C can be incorporated into gel formulations or protective coatings to support dental implant osseointegration and accelerate healing following periodontal surgery [13]. Supplementing with oral vitamin C aids in postoperative healing for individuals with chronic periodontitis and those receiving dental implants, such as Bio-Oss collagen transplantation or guided bone regeneration [22]. Even with proper oral care, a vitamin C deficiency can cause oxidative stress, leading to tissue damage and being linked to gum bleeding [22,43]. Chapple et al. [43] found that people with serum vitamin C levels below 8.52 mmol/L had a significantly higher frequency of severe periodontitis than people with higher vitamin C concentrations [44]. Figure 2 illustrates vitamin C’s role in osseointegration, collagen formation, and immune modulation by enhancing B and T cell proliferation, increasing antiviral factors in macrophages and dendritic cells, and reducing inflammatory cytokines to support periodontal health.

Vitamin D: The liver first converts calciferols, a fat-soluble vitamin that is necessary for controlling calcium absorption, to 25-hydroxyvitamin D (25(OH)D), which the kidneys then change into 1,25-dihydroxyvitamin D (1,25-(OH)2D), which is its active form [45]. Enhancing the absorption of key minerals, including Ca^2+^, iron (Fe^2+^/Fe³⁺), Mg^2+^, potassium (K^+^), and Zn^2+,^ requires vitamin D, which is mainly obtained from fish, fortified foods, and mushrooms [20]. There are two main types of vitamin D: vitamin D_2 _(ergocalciferol) and vitamin D_3 _(cholecalciferol). The UK National Health Service recommends consuming 10 µg of vitamin D daily [46,47].

It is well known that vitamin D is crucial for the immune system and bone homeostasis. It provides a strong biological basis for the hypothesis that its deficiency may adversely affect periodontal health [45,48]. Vitamin D stimulates the synthesis of cytokines, improves immune cell proliferation, encourages monocyte differentiation into macrophages, and helps protect against infections brought on by pathogens [24]. Insufficient blood Ca²⁺ levels impair proper bone tissue calcification, contributing to the development of rickets and osteomalacia [12]. Gingival inflammation and an increased risk of tooth loss are linked to vitamin D [10]. A case-control study demonstrated that low vitamin D levels are associated with a higher likelihood of PD among pregnant women [49]. Several studies have shown that vitamin D's anti-inflammatory qualities assist in lowering the prevalence of gum and PD [12]. Beyond its role in calcium regulation, vitamin D may influence the immune system, providing anti-inflammatory and antimicrobial benefits that could alter the progression and severity of PD [50]. Too much vitamin D can cause hypoplasia and other dental problems, including pulp stones [51]. Low blood vitamin D levels during pregnancy seem to be associated with enamel hypoplasia [10]. Vitamin D is recognized for its anti-inflammatory effects, with studies linking low levels to periodontitis, especially in middle-aged and older adults. Starting vitamin D supplementation in middle age may help prevent PD progression, as aging raises the risk of bone health issues such as osteoporosis [5].

Vitamin E: Alpha-tocopherol, discovered in 1922 for its positive effects on rat reproduction [5,52], is a plant-derived, lipid-soluble substance (e.g., γ-, β-, α- and δ-tocopherols), as well as their corresponding tocotrienols, which make up its eight constituents [5]. Eight naturally occurring, lipid-soluble antioxidants comprise the vitamin E complex, including four tocopherols and four tocotrienols [20]. Tocopherols are viscous at ambient temperature and insoluble in water, although they dissolve in ethanol and aprotic solvents. The most prevalent and active vitamin E in humans is α-tocopherol [5,53]. Nuts, fruits, seeds, forfeited cereals, and vegetable oils are the primary dietary sources of vitamin E [20,49]. As an antioxidant, vitamin E works with adipose tissue and cell membranes to reduce the generation of reactive oxygen species (ROS) [33]. Vitamin E supplementation supports the recovery and regeneration of periodontal tissues [33,54]. Supplementing with vitamin E may help repair and safeguard against pulmonary inflammation [49]. Vitamin E stabilizes the structure of cell membranes by stopping free radical reactions [13,55]. The liver is essential for controlling vitamin E levels through its metabolic processes [55]. The association between periodontitis and mixed dietary vitamins is significantly harmed by vitamin E [56].

Vitamin K: Phylloquinone and menaquinones, which are plant-derived fat-soluble vitamins, can be found in various fruits, vegetables, and oils, such as avocado, green grapes, lettuce, broccoli, and kiwi [5]. It belongs to a class of biomolecules crucial for producing proteins vital for blood clotting and regulating the body's hemostatic processes [5,57]. Biologically, it regulates calcium metabolism, oxidative stress, cell growth and proliferation, and inflammation [58]. Vitamin K is crucial in producing vitamin K-dependent proteins (VKDPs) [59]. These include seven proteins critical for blood clotting (factors II, VII, IX, and X, protein S, protein C, and protein Z) and four proteins in the transmembrane Gla family: osteocalcin (OCN) (bone Gla protein), matrix Gla protein, growth arrest-specific six protein (Gas6), and protein S [5]. Insufficient vitamin K levels may lead to decreased bone density [12]. By enabling the posttranslational modification of glutamate residues in coagulation factors, vitamin K improves its ability to bind to phospholipid membrane surfaces, thereby aiding in the prevention of gingival bleeding [60,61]. Vitamin K inhibits bone resorption by stimulating osteoblast proliferation, differentiation, and function while preventing Fas-mediated apoptosis [5]. Furthermore, vitamin K_2 _boosts alkaline phosphatase activity, encourages the expression of bone-building markers such as OCN, and aids in transforming osteoblasts into osteocytes [5,62]. Vitamin K deficiency is frequently associated with periodontal issues, including gum bleeding and the weakening of tooth clinical attachment [12,60].

Minerals: Minerals are vital inorganic substances that form crystals, with their concentrations in the human body and dietary consumption differing considerably. While some minerals are essential for enzyme function, others contribute to fluid balance, bone development, hormone production, nerve signal transmission, muscle movement, and protection against harmful free radicals [1]. About 4-5% of the body's weight is composed of trace elements [12]. Minerals are essential for maintaining a stable heart rhythm, aiding nerve transmission and muscle contraction, and balancing the body's acid-base levels [63-65]. Major minerals are needed in more significant quantities than 100 mg daily, while trace minerals are required in lesser amounts, less than 100 mg daily [65,66].

Macro-minerals (macro-elements) consist of Ca²⁺, phosphorus (PO₄³⁻), Mg²⁺, sodium (Na⁺), K⁺, and chloride (Cl⁻) [12,65]. Trace elements (micro-elements) encompass Fe²⁺/Fe³⁺, I⁻, Zn²⁺, selenium (Se²⁻), cobalt (Co²⁺/Co³⁺), chromium (Cr³⁺), copper (Cu⁺/Cu²⁺), fluorine (F⁻), molybdenum (Mo), and several others [12,65].

Localized Ca²⁺ application promotes osseointegration, while supplementation with Ca²⁺ and magnesium Mg²⁺ enhances the effectiveness of nonsurgical periodontal therapy [1]. F⁻ protects teeth and prevents dental decay [1]. Ca²⁺ is crucial for oral health, contributing to bone and tooth development; about 99% of the calcium in the body is stored as hydroxyapatite in the teeth and bones, while the remaining 1% resides in bodily fluids and soft tissues. It plays a vital role in cellular signaling and processes, with extracellular levels regulated by hormones to maintain homeostasis, support bone mineralization, and aid muscle function [17]. Intracellular calcium is essential for membrane permeability, neurotransmitter release, and critical cellular functions such as proliferation, differentiation, and apoptosis [67]. Ca²⁺ supplements may improve periodontal treatment outcomes, as sufficient intake from diet or supplements is associated with a reduced risk of tooth loss and better long-term retention [68]. Fe²⁺/Fe³⁺ is essential for metabolic enzyme function and hemoglobin production. A deficiency may result in anemia, triggering oxidative stress and disrupting the antioxidant-defense system, adversely affecting periodontal health [17]. Fe²⁺/Fe³⁺ is essential for gum health, and its deficiency weakens the defense against periodontal pathogens [69].

Zn²⁺ is essential for tissue repair, cell growth, and development. It also helps keep cell membranes stable. Acting as a cofactor for more than 300 enzymes, it is essential to control how proteins, lipids, and nucleic acids are metabolized while enhancing immune defense, providing antioxidant benefits, and supporting crucial metabolic activities [70]. Fe²⁺/Fe³⁺ and Zn²⁺ have antioxidant effects that support periodontal health, with zinc also helping to reduce the severity of periodontitis associated with diabetes [1]. It is crucial for bone mineralization, blood clotting, other homeostatic processes, and injury repair, all of which are linked to periodontal health [23].

K⁺, the primary intracellular cation, is essential for enzymatic activity, metabolic processes, cellular growth, and DNA synthesis [12]. Insulin, alkalosis, and catecholamines regulate potassium balance, with nearly 90% of dietary intake being absorbed and later eliminated through urine. Low levels (hypokalaemia) can lead to muscle weakness, slow digestion, and heart rhythm disturbances [17]. Mg²⁺ supports metabolism, bone formation, reproduction, and immune function. It also collaborates with vitamin K in blood clotting, while its deficiency can impair bone density and formation [17,71]. Kim et al. discovered a negative correlation between periodontal health and plasma manganese levels, regardless of systemic conditions, oral hygiene, sociodemographic factors, or overall oral health [71].

Se²⁻ supports periodontal health with its antioxidant effects, while selenoproteins and Se²⁻ prevent autoimmunity, immune regulation, and chronic inflammation [72]. Despite being regarded as hazardous trace elements, lead and mercury may have some advantages when present in small amounts. Lead exposure can disrupt immune function and bone metabolism, heightening the risk of periodontitis, while long-term exposure may damage the periodontal ligaments, gums, and alveolar bone [73]. Table 1 presents daily mineral requirements by age, pregnancy status, and sources for key minerals such as Na^+^, Ca^2+^, F⁻, PO₄³⁻, Fe²⁺/Fe³⁺, Mg^2+^, K^+^, Mo, I⁻, Cr³⁺, Zn²⁺, Cu⁺/Cu²⁺, and Se²⁻.

**Table 1:** Daily recommended dose of minerals with sources. Table Credit: Dhruvi Doshi

Variables	Age	Pregnancy	Non-pregnancy	Source
Mg²⁺ [63]	14-18 years, 19-30 years, 31-50 years	400 mg/day, 350 mg/day, 360 mg/day	310-420 mg/day	Meat, grains, bread, beans, and vegetables
Ca²⁺ [64]	14-18 years, 19- 50 years	1300 mg/day, 1000 mg/day	1000 mg/day	Milk, cheese, tofu, yogurt, green leafy vegetables, and fish
PO₄³⁻ [74]	14-18 years, 19-50 years	1250 mg/day, 700 mg/day	700 mg/day	Grains, meat, eggs, milk, milk products, nuts, and beans
Na⁺ [74-76]	14-50 years	1500 mg/day	1500-2300 mg/day	Salt, cheese
K⁺ [74,75,77]	14-50 years	4700 mg/day	4700 mg/day	Fruits, vegetables, nuts, milk, yogurt, fish, and meat
Mo [76]	14-50 years	50 mcg/day	45 mcg/day	Milk, meat, bread, grains, and legumes
Fe²⁺/Fe³⁺ [77]	14-50 years	27 mg/day	8-18 mg/day	Tofu, fortified cereals, green leafy vegetables
Cu⁺/Cu²⁺ [77]	14-50 years	1 mg/day	900 mcg/day	Whole grains, dark chocolate, nuts, and seeds
I⁻ [78]	14-50 years	220 mcg/day	150 mcg/day	Iodized salt, fish, seafood, dairy products, eggs
Zn²⁺ [78]	14-18 years, 19-50 years	12 mg/day, 11 mg/day	8-11 mg/day	Dark green and dark yellow vegetables, meat, shellfish, eggs, grains, peanuts, dairy products, and whole grains
Se²⁻ [79]	14-50 years	60 mcg/day	55 mcg/day	Plant foods (such as cereals, nuts, garlic, and radishes), seafood, chicken, eggs, kidney, and liver
Cr³⁺ [80]	14-18 years, 19-50 years	29 mcg/day, 30 mcg/day	25-35 mcg/day	Liver, nuts, and whole grains
F⁻ [81]	14-50 years	3 mg/day	3-4 mg/day	Marine fish, particularly those with edible bones and tea

Since Na⁺ makes up a large portion of extracellular fluid, it is the body's predominant electrolyte and is essential for fluid balance and neuronal transmission [75]. Better periodontal health has been linked to a greater intake of micronutrients, such as Zn^2+^, fiber, and omega-3; higher energy consumption; advanced education levels; and a reduced occurrence of non-transmissible chronic diseases (NTCD) [23]. The immune system and defense against oxidative stress depend heavily on zinc, copper, and magnesium [23]. According to Dattani et al., insufficient calcium intake in the diet could contribute to the progression of severe PD [12]. Cu⁺/Cu²⁺ is recognized for its anti-inflammatory properties [49]. Inadequate calcium intake in the diet is associated with a higher chance of getting PD and could worsen it [51].

Role of Micronutrients in PD

Gingivitis, or gum inflammation, and periodontitis, or inflammation of the deeper periodontal tissues, are the two primary disorders that make up PD [49]. The mildest stage of PD, gingivitis, is curable, while periodontitis irreversibly damages bone and connective tissue [49].

Antioxidant Micronutrients and Periodontal Health

Various antioxidant micronutrients are present in the human diet, including vitamin E (α-tocopherol), vitamin A (carotenoids and β-carotene), vitamin C (AA), melatonin, and glutathione. Research suggests that these nutrients may help reduce inflammation in periodontal tissues by counteracting damage caused by ROS [13]. Consuming a nutrient-rich diet abundant in antioxidants and anti-inflammatory compounds has been strongly linked to a reduced likelihood of developing periodontitis and a slower progression of the disease [5]. Higher consumption of fruits, vegetables, omega-3 fatty acids, and vitamins A, C, and E has been linked to improved population health in terms of periodontal tissue [82].

Vitamin A and PD

A deficiency in vitamin A weakens antigen responses, reduces lymphocyte production, lowers antibody and immunoglobulin levels, increases bacterial adhesion, and impairs immune cell differentiation, ultimately compromising immune function [12,47]. Following in vitro treatment with retinoic acid (RA), gingival and periodontal fibroblasts exhibited increased expression of keratinocyte growth factor (KGF), which plays a vital role in epithelial wound healing, oral epithelial tissue maintenance, and development [83]. Enhancing highly porous three-dimensional hydroxyapatite scaffolds with vitamins and a biotin-avidin combination improved adhesion and stronger attachment of periodontal ligament fibroblasts [84]. Low cobalamin levels were associated with more significant CAL and increased tooth loss [5].

Effect of Vitamin B Complex on Periodontal Condition

**Figure 3:**
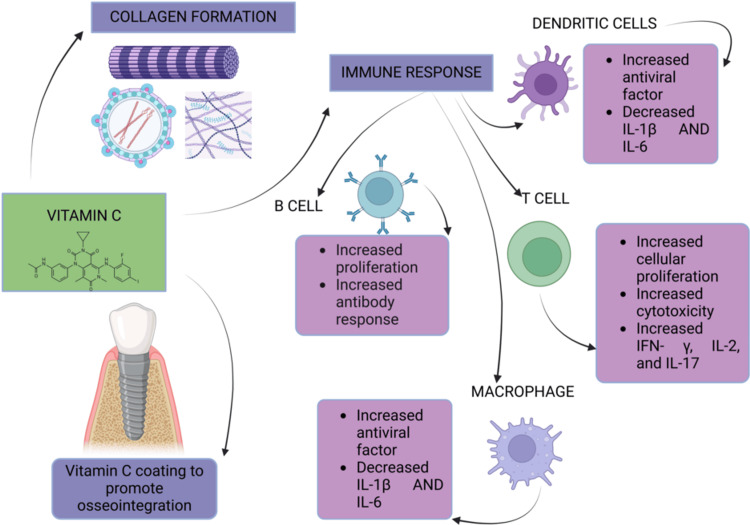
Effect of vitamin B complex on the periodontium. Notes: This image was created with BioRender [16] premium version, with an agreement license number PE28293JY1. Illustration Credit: Dhruvi Doshi

Findings suggested that individuals using FA were more likely to be diagnosed with PD. FA, a B-complex vitamin, is involved in cell division, immunological cell function, and cellular metabolism [85]. According to one research conducted by Shetty et al., vitamin B supplementation can accelerate postsurgical healing [1]. In a prospective population study, Zong et al. [38] examined the connection between blood vitamin B_12_levels and the development of periodontitis. Their findings revealed that a decrease in the Parkinson's indication was associated with elevated blood levels of vitamin B_12_-Parkinson's illness [38]. Serum folate levels and periodontal health were evaluated using the 2001-2002 National Health and Nutrition Examination Survey (NHANES) information. Findings revealed no independent association between PD and low serum folate, though a statistically significant negative correlation was identified [12]. Figure 3 illustrates the role of the vitamin B complex in periodontal health, highlighting how different B vitamins (B_1_, B_6_, B_2_, B_3_, B_9_, and B_12_) contribute to inflammation reduction, wound healing, collagen synthesis, immune regulation, oral tissue maintenance, and prevention of periodontal lesions and bone loss.

Role of Vitamin C in Periodontal Health

By promoting the differentiation of periodontal ligament progenitor cells, vitamin C contributes significantly to slowing the progression and preventing the onset of PD [44]. AA is essential for collagen synthesis. It supports the hydroxylation of proline and lysine while activating prolyl hydroxylase in procollagen [86,87]. AA strengthens newly formed collagen, allowing it to stretch without tearing [87]. Despite the paucity of research on its role in reducing gum pigmentation in dentistry, AA is well recognized as a safe and effective component of minimally invasive treatments [88].

Role of Vitamins D, E, and K

After initial treatment, maintaining periodontal health has been associated with adequate vitamin D consumption [5]. In cells exposed to toxins, vitamin E is more effective than vitamin C at increasing cell survival, migration, and proliferation while decreasing apoptosis [89]. Studies have shown that applying UV-activated vitamin D precursors and vitamin E coatings to implant surfaces reduces inflammation and extracellular matrix degradation in human gingival fibroblast (HGF) cultures. Collagen III α1 and fibronectin mRNA expression increased as a result, and TIMP-1 mRNA and protein levels increased, while IL-8 mRNA levels decreased [90]. Shetty et al. concluded that vitamin D deficiency might delay postsurgical healing, whereas local application can support healing and improve osseointegration [1]. Vitamin D supports immune regulation by controlling cytokine and chemokine production and affecting T and B lymphocytes, helping maintain immune balance and prevent excessive gingival inflammation. It also has anti-inflammatory effects by lowering the levels of pro-inflammatory cytokines, including IL-6 and TNF-α [17].

**Figure 4:**
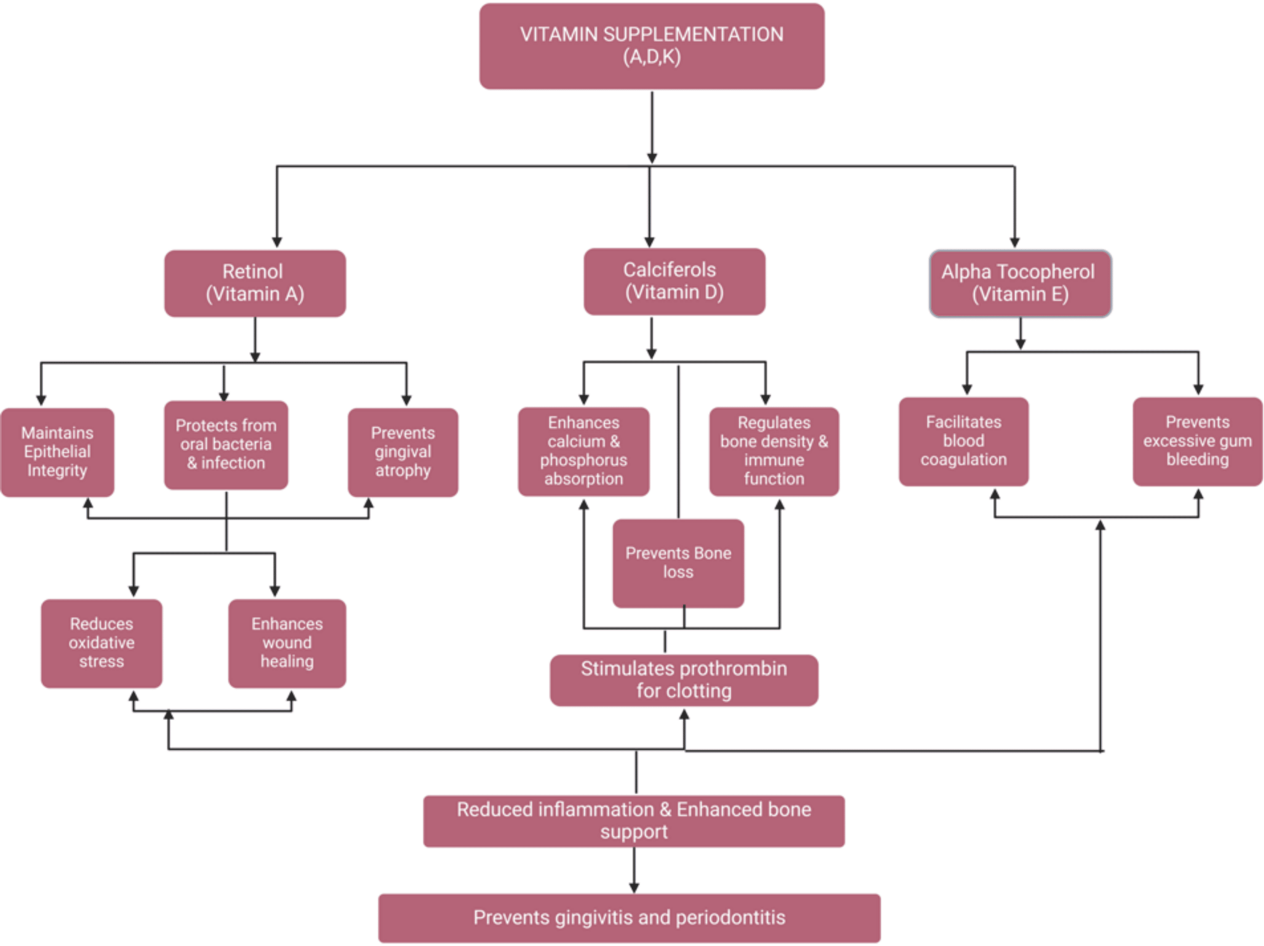
Effect of different types of vitamin supplementation on the periodontium. Notes: This image was created with BioRender [16] premium version, with an agreement license number EK282A3X2F. Illustration Credit: Dhruvi Doshi

By restoring redox balance, reducing inflammation, and promoting wound healing, vitamin E plays a supportive role in maintaining periodontal health [85]. Shetty et al. reported that vitamin E deficiency can lead to gingival bleeding, whereas its adjunctive supplementation has no documented impact on periodontal therapy [1]. According to a systematic review, people with periodontitis had a negative correlation between higher blood α-tocopherol levels and PPD, while gamma-tocopherol showed a modest correlation with lower PPD [17]. The highest biomass was observed in the medium supplemented with hemin and vitamin K, indicating that vitamin K influenced biomass production [5]. Figure 4 illustrates the impact of vitamin supplementation (A, D, K, and E) on periodontal health, emphasizing their roles in maintaining epithelial integrity, supporting immune function, enhancing bone density, promoting blood coagulation, and reducing inflammation to prevent gingivitis and periodontitis.

Minerals and Periodontal Health

**Figure 5:**
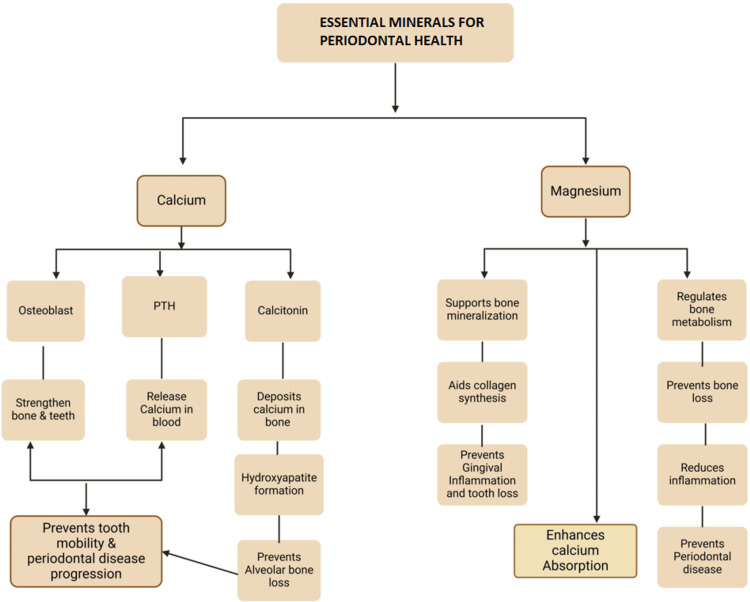
Effect of different minerals on the periodontium. Notes: This image was created with BioRender [16] premium version, with an agreement license number RC282AJH48. Illustration Credit: Dhruvi Doshi

Inadequate intake of PO₄³⁻, Ca²⁺, and Mg²⁺ could trigger impaired absorption, accelerate bone resorption, heighten tooth mobility, increase the likelihood of premature tooth loss, and raise the risk of hemorrhage [51]. Figure 5 illustrates the vital role of Ca^2+ ^and Mg^2+^ in periodontal health, emphasizing their contributions to bone strength, mineralization, collagen synthesis, calcium absorption, and inflammation control to prevent tooth mobility, bone loss, and PD progression.

**Figure 6:**
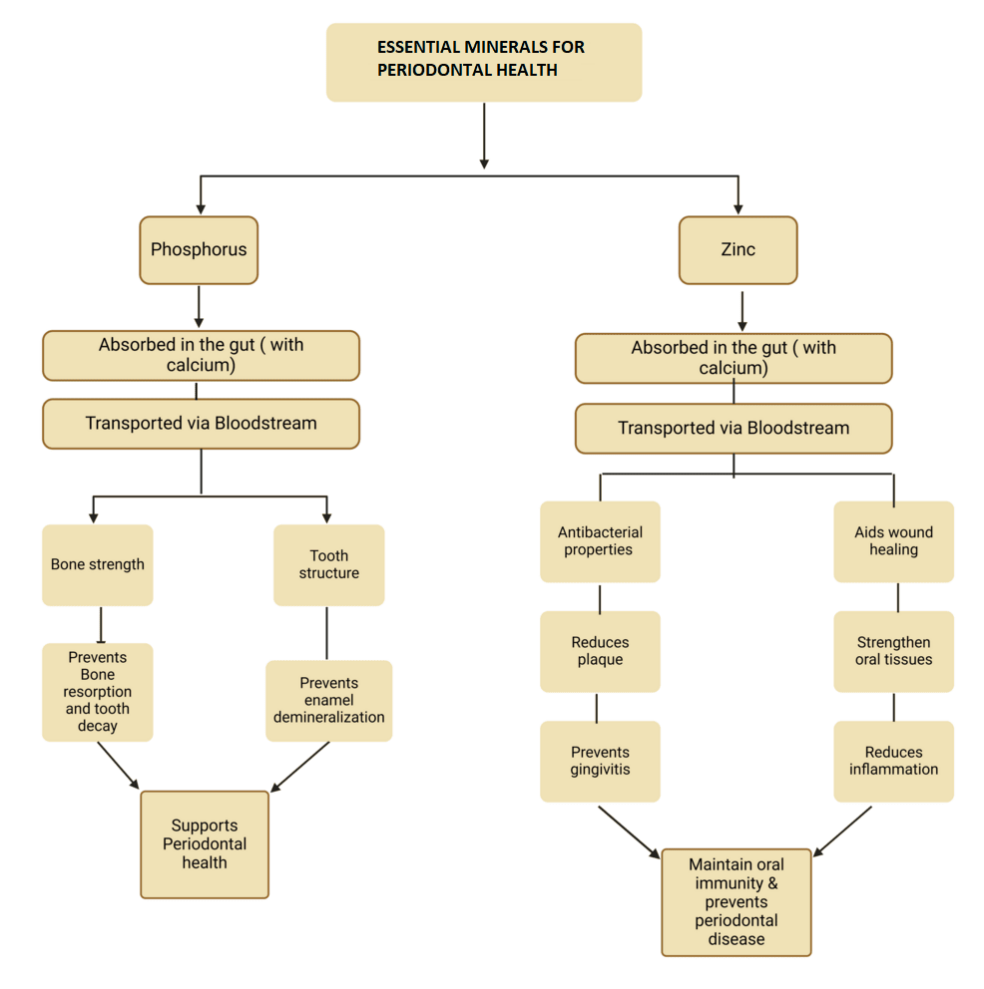
Effect of different minerals on the periodontium. Notes: This image was created with BioRender [16] premium version, with an agreement license number DE282ANMB8. Illustration Credit: Dhruvi Doshi

Iron deficiency can lead to angular cheilosis, pallor, a burning tongue, glossitis, filiform papillae atrophy, and candidiasis, all due to weakened immune function [51]. In the present study, patients taking iron supplements had a reduced risk of developing PD [85]. Studies have indicated that macronutrients (docosahexaenoic acid (DHA) and eicosapentaenoic acid (EPA), a long-chain omega-3 fatty acid that is present in fatty fish) and micronutrients (vitamins D and B) can aid in patients' recuperation following periodontal therapy [19]. Anemic patients with chronic periodontitis show more bleeding on probing, more considerable attachment loss, and deeper pockets than those without anemia [69]. Figure 6 shows how phosphorus and zinc support periodontal health by strengthening bones and teeth, aiding immunity, healing wounds, and reducing inflammation to prevent bone loss, enamel erosion, plaque, gingivitis, and disease.

Disruptions in Ca^2+^ homeostasis during PD can exacerbate inflammatory responses to pathogens, leading to periodontal tissue damage. Research indicates that sufficient calcium intake may reduce the likelihood of periodontitis by approximately 20% while lowering the risk of disease progression and attachment loss, independent of key factors such as smoking and blood sugar levels [17]. Essential for maintaining healthy gums, Zn^2+^ aids in enzyme activity, the periodontal tissues' structural soundness, and protein stabilization. A deficiency in this mineral can lead to gingival inflammation through multiple biological mechanisms [70].

Role of Nutrition in Periodontal Health in Low- and Middle-Income Countries

Malnutrition is substantial and impacts periodontal health, particularly in India, where undernutrition remains a concern. It weakens immunity, delays tissue repair, and raises the risk of PD. Studies in Indian children associate malnutrition with impaired tissue homeostasis and lower resistance to microbial biofilms, contributing to poor oral health [91]. Inadequate nutrition has a profound effect on periodontal well-being, especially in middle- and low-income nations where malnutrition is prevalent. It compromises immune function, delays tissue healing, and heightens susceptibility to infections, including diseases affecting the periodontium [92].

Research published in the International Dental Journal associated deficiencies in vitamins A, B_1_, C, and E, along with inadequate levels of phosphorus, folate, and iron, with a greater severity of PD. These results highlight the critical role of nutritional interventions in preserving periodontal health, especially in low- and middle-income countries where malnutrition remains widespread [93]. Research conducted in southwestern India has indicated that individuals with periodontitis, especially those with diabetes, exhibit higher copper levels compared to those without gum disease [94]. In older adults, malnutrition is associated with oral pain, chewing difficulties, tooth loss, and worsening periodontal health [95]. Poor nutrition speeds up PD progression. A review on malnutrition and oral health revealed that undernourished individuals face rapid disease advancement, starting in the gums and extending to the periodontal ligament and alveolar bone [96]. These findings demonstrate how important diet is to maintaining periodontal health in India. Preventing and treating PD depends on improving eating habits and implementing public health initiatives.

Overall Influence of Micronutrients on Oral Health

Effect of vitamins on oral health: Vitamins play a vital role in oral health by strengthening gums, bones, and the immune system. Vitamin C supports collagen production, protecting against gum disease [40]. Vitamin D boosts Ca^2+^ absorption for strong teeth and bones [20]. Vitamin A can enhance the osteogenic differentiation, proliferation, and migration of human periodontal ligament cells [22]. B-complex vitamins prevent mouth sores [85], and Vitamin K helps preserve bone density, reducing PD risk [58].

Impact of microelements on oral health: Microelements such as Ca^2+^, Zn^2+^, Fe^2/3+^, and Mg^2+^ play a crucial role in maintaining oral health. Ca^2+^ strengthens teeth and bones, reducing the risk of decay and PD [17]. Zn^2+^ aids in wound healing and supports gum immunity [23]. Fe^2/3+^ deficiency may lead to oral ulcers and inflammation [17], while Mg^2+^ helps maintain enamel integrity, preventing cavities and gum disease [1]. A balanced intake of these microelements promotes overall oral well-being.

Influence of vitamin and micro-element deficiency on oral health: A paucity of vitamins and microelements can considerably harm oral health. A lack of vitamin C weakens gums, leading to bleeding and slow healing [6]. Vitamin D deficiency results in weak teeth and bone loss [1]. A lack of Zn^2+^ raises possibilities, resulting in delayed gum healing and increased inflammation [70].

How micronutrients prevent periodontal tissue inflammation: Micronutrients play a key role in reducing inflammation by strengthening the immune system and minimizing oxidative stress. Vitamin C is an antioxidant that supports tissue repair and reduces gum swelling [40]. Vitamin D helps regulate immune responses, stimulates the synthesis of cytokines, and prevents excessive inflammation [24]. Zn^2+^ and Mg^2+^ promote wound healing [70].

Future Research Perspective

Micronutrients play a crucial role in periodontal health, yet research gaps remain. Future studies should investigate the role of specific micronutrients, such as vitamin D, zinc, and omega-3 fatty acids, in disease progression and tissue regeneration. Molecular and genetic research can reveal how vitamins, minerals, and bioactive compounds regulate inflammation and immune responses. A key area of interest is the combined effects of multiple micronutrients. While most studies focus on individual nutrients, real-world diets involve complex interactions. Investigating combinations such as vitamin D, Zn^2+^, omega-3 fatty acids, and calcium could provide new insights. Clinical trials are needed to determine optimal supplementation dosages and treatment durations. Personalized nutrition, guided by nutrigenomics, may help tailor dietary recommendations. Developing micronutrient-enriched oral care products, such as mouth rinses and gels, could offer adjunctive benefits in periodontal therapy.

Limitations of This Narrative Review

This narrative review on micronutrients and periodontium has limitations. It relies on existing literature, which may contain biases and inconsistencies. Most studies are observational, lacking substantial clinical trials to confirm causation. Variations in methodologies, sample sizes, and intervention durations hinder comparisons. Genetic, lifestyle, and environmental factors affecting nutrient absorption are not considered. The absence of standardized dosages limits clinical application, highlighting the need for further controlled trials.

## Conclusions

To summarize, periodontitis is a long-term inflammatory disease that advances slowly, primarily due to a disruption between antioxidant protection and the repair mechanisms controlled by ROS. When this balance tips toward increased free radical production, it destroys cells and tissue. Therefore, maintaining adequate antioxidant concentrations in oral fluids is essential to protecting against such damage and promoting optimal oral health. Antioxidants obtained through diet contribute significantly to oral health and may impact PD management, leading to improved clinical results. Nutritional status is strongly associated with the host's effectiveness of the host's immune response. Adequate nutrition is crucial for maintaining healthy tissues, supporting immune function, and preventing PD. Specific nutrients are needed to repair damaged tissue and promote healing once gingivitis or periodontitis occurs. Adequate vitamin C intake is essential for preventing gum bleeding, while insufficient vitamin K levels may contribute to periodontal issues, including gum hemorrhage and the weakening of tooth attachment. A lack of these essential nutrients may impair immune response, increasing susceptibility to oral infections and worsening their severity. Specific vitamins have been closely linked to the occurrence and progression of PD. Vitamin deficiencies can either contribute to a patient’s increased vulnerability to PD or result from active PD. Adequate intake of dietary antioxidants is essential for maintaining oral health and effectively managing PD, leading to improved clinical outcomes. Higher antioxidant levels are associated with better periodontal health, highlighting their role in protecting tissues from oxidative damage caused by free radicals. This reinforces the crucial role of antioxidants in supporting periodontal well-being.
